# Genetic-based prediction of disease traits: prediction is very difficult, especially about the future^†^

**DOI:** 10.3389/fgene.2014.00162

**Published:** 2014-06-02

**Authors:** Steven J. Schrodi, Shubhabrata Mukherjee, Ying Shan, Gerard Tromp, John J. Sninsky, Amy P. Callear, Tonia C. Carter, Zhan Ye, Jonathan L. Haines, Murray H. Brilliant, Paul K. Crane, Diane T. Smelser, Robert C. Elston, Daniel E. Weeks

**Affiliations:** ^1^Center for Human Genetics, Marshfield Clinic Research FoundationMarshfield, WI, USA; ^2^Department of Medicine, School of Medicine, University of WashingtonSeattle, WA, USA; ^3^Departments of Human Genetics and Biostatistics, Graduate School of Public Health, University of PittsburghPA, USA; ^4^Sigfried and Janet Weis Center for Research, Geisinger Health SystemDanville, PA, USA; ^5^Subsidiary of Quest Diagnostics, Discovery Research, Celera CorporationAlameda, CA, USA; ^6^Department of Biological Sciences, University of PittsburghPittsburgh, PA, USA; ^7^Biomedical Informatics Research Center, Marshfield Clinic Research FoundationMarshfield, WI, USA; ^8^Department of Epidemiology and Biostatistics, Case Western Reserve School of MedicineCleveland, OH, USA

**Keywords:** predictive model, genetic risk, human genetics, prognosis, clinical utility

## Abstract

Translation of results from genetic findings to inform medical practice is a highly anticipated goal of human genetics. The aim of this paper is to review and discuss the role of genetics in medically-relevant prediction. Germline genetics presages disease onset and therefore can contribute prognostic signals that augment laboratory tests and clinical features. As such, the impact of genetic-based predictive models on clinical decisions and therapy choice could be profound. However, given that (i) medical traits result from a complex interplay between genetic and environmental factors, (ii) the underlying genetic architectures for susceptibility to common diseases are not well-understood, and (iii) replicable susceptibility alleles, in combination, account for only a moderate amount of disease heritability, there are substantial challenges to constructing and implementing genetic risk prediction models with high utility. In spite of these challenges, concerted progress has continued in this area with an ongoing accumulation of studies that identify disease predisposing genotypes. Several statistical approaches with the aim of predicting disease have been published. Here we summarize the current state of disease susceptibility mapping and pharmacogenetics efforts for risk prediction, describe methods used to construct and evaluate genetic-based predictive models, and discuss applications.

## Introduction and background

Multiple lines of evidence strongly support the notion that the large majority of common, chronic diseases have complex causes. Environmental components such as infection, caloric flux, and chemical exposure, along with heritable elements such as DNA variants, methylation patterns, and epigenetic RNA effects, are interacting co-conspirators resulting in common diseases. In this background of convoluted and entangled etiology, discovery and use of disease predisposing alleles present a considerable challenge to the human genetics community (Clerget-Darpoux and Elston, 2013). Recent technological advances in high-throughput genotyping, RNA expression, and massively parallel sequencing have accelerated interrogation of genetic variation for the purpose of understanding human disease and drug response. Among the more important uses of these discoveries is providing detailed, mechanistic insight into the molecular pathogenesis of disease states. The two primary avenues of utilizing this explosion in genetic information for the purpose of improving clinical practice are in (1) drug development stemming from the identification of molecular targets and (2) the prediction of disease susceptibility, pharmacogenetic response, and disease severity/trajectory (Khoury et al., 1985; Holtzman and Marteau, 2000; Evans and Relling, 2004). Although only a small minority of current pharmaceuticals originated directly from genetic findings serving as drug targets, the list is expanding and includes inflammatory cytokine-based monoclonal antibodies and targeted cancer therapeutics, among others. These therapeutics often target specific biochemical pathways to improve clinical treatment, often with a reduction in adverse reactions. Disease prediction and diagnosis with genetic testing is a broad field with diverse applications, ranging from karyotyping for chromosomal abnormalities to enhancement of disease risk profiles using single nucleotide polymorphisms (SNPs) previously found to be disease-susceptibility markers, such as *HFE* missense polymorphisms which can lead to hemochromatosis, or the variants in the tumor suppressors *BRCA1* and *BRCA2* that increase risk to breast and ovarian cancers. Clinical genetics testing can provide physicians with an additional tool for better diagnosis and improved medical care.

Much of the variation in disease course, severity, and response to medication is reflective of the underlying allelic repertoire existing in each individual, offering the opportunity for genetics to facilitate early treatment, preventative medicine, preemptive selection of efficacious drugs, and more accurate estimation of risk for those thought to be at intermediate risk using traditional factors. As the cost and complexity of medical care escalates, the promise of human genetics to provide directly actionable, individualized information to address impediments to optimal and cost-effective medical practice carries increasing weight and urgency (Chen and Snyder, 2013). This review has multiple aims: (1) provide a brief overview of the current state of human disease mapping as this provides the foundational knowledge for genetic-based disease prediction, (2) describe the process of disease prediction in a simple probabilistic framework detailing the general qualities of clinically useful predictive models and also detailed examples, (3) provide an overview of the basic classes of genetic-based prediction models and measures of prognostic utility, and (4) illustrate the application of genetic-based predictive models to data from biobanks and prospective cohorts.

Identification of replicated susceptibility variants provides considerable material for understanding biochemical pathways that govern diseases, particularly when the variants reside within the coding or regulatory regions of well-understood genes and are validated by functional studies (Manolio, 2010). Unfortunately, many disease-associated variants are located in regions of the genome that have not yet been functionally characterized. Indeed, 39% of the National Human Genome Research Institute (NHGRI) Genome-Wide Association Study (GWAS) catalog SNPs are annotated as intergenic and more than 36% are reported as intronic (Welter et al., 2014). The genes and pathways discovered can become targets for pharmaceutical intervention, especially when integrated with corroborating studies from disease models, signal transduction experiments, bioinformatics, and protein biochemistry. Examples of using specific genes or their products as pharmaceutical targets have rapidly accumulated over the past decade and include mipomersen, an antisense therapeutic targeting *APOB* RNA for the treatment of hypercholesterolemia (Raal et al., 2010), ivacaftor which targets the G551D mutation in *CFTR* found in approximately 4% of individuals with cystic fibrosis (Ramsey et al., 2011), inflammatory cytokines and their receptors (e.g., IL-1β, IL-12/23p40, IL-17A, IL-6R) (e.g., Krueger et al., 2007), other immune cell signaling proteins (e.g., CTLA-4, CD30), a variety of tumorigenesis genes that harbor somatic variants useful for individualized cancer treatment (e.g., *BRAF, KRAS*, and *EGFR*), and lipid transfer proteins (e.g., CETP, PCSK9), among many others. As more human genetics studies are conducted, the number of these druggable targets will expand. While the use of genetic results in pharmaceutical development is impressive, some of the most highly touted uses of genetic susceptibility data have been the accurate prognosis of diseases (e.g., Mendelian and oligogenetic disorders, such as Tay-Sachs disease, phenylketonuria, Charcot-Marie-Tooth, or rare ciliopathies including polycystic kidney disease and Bardet-Biedl syndrome), or other areas that impact medical decisions, such as choice of drug, selection of dose, avoidance of side-effects, or determining the optimal intensity of clinical monitoring. Unlike identifying potential drug targets, genetic-based prediction models may serve a clinical purpose in advance of precise identification of the functional motifs and molecular mechanisms that drive genetic association/linkage signals. Instead, the utility of predictive models is derived primarily from the correlation patterns—provided that these are robust across intended populations. However, the strength and robustness of the correlation are critical for a genetic prediction model to be clinically useful.

As clinical decisions are specific to individuals, physicians aim to assess the probability of medical traits for each patient. This is a dynamic process where physicians update assessments as additional relevant information, such as laboratory tests (both genetic and non-genetic), or changes in physiology become known. In this way, clinical decisions are informed as variation in an individual's risk to disease, severity of disease and response to medication are progressively revealed. Thus, results from clinical tests, including genetic-based predictive models, are useful when they more accurately discern the likelihood of the medical trait (e.g., disease occurrence or response to medication), compared to the pre-test assessment. For example, if a physician had estimated that a patient had a 40% chance of having a particular disease prior to the results of a clinical test, and the 40% prediction remains unaltered following the results of the test, then the clinical test and new prediction may be of little value. Further, whether the magnitude of this posterior-prior probability departure carries clinical utility depends on the specific application. As an illustration of this process, suppose a patient is referred to a rheumatologist. Prior to the visit, the rheumatologist may not have sufficient information to modify the assessment of the probability that the individual has, for example, rheumatoid arthritis (RA). Upon learning that the patient self-reported symptoms of symmetric sore joints that are partially remediated by non-steroidal anti-inflammatory medication, the rheumatologist proceeds to update the probability of RA and of other conditions. Some diseases would increase in their likelihood, while others would decrease from their initial values. Following a standard evaluation of the classification criteria for rheumatoid arthritis assessing joint involvement, serology, acute-phase reactants, and symptom duration (Aletaha et al., 2010), the rheumatologist proceeds to further update the probability that the patient suffers from RA. Subsequent testing of a genetic panel of known RA-susceptibility markers, including polymorphisms within *HLA-DRB1, PTPN22, STAT4, CTLA4, TRAF1, CD40*, etc., may further modify the posterior probability. This additional updated posterior probability may be particularly useful in situations where a definitive diagnosis was not available with non-genetic approaches alone. This is, of course, neither a new nor complete account of the diagnostic process, but it underscores the general nature of many medical decisions, where accumulation of information typically results in increasing accuracy in the appraisal of a medical trait probability for an individual. The process of serial refinement based on accumulating data is a hallmark of the diagnostic process and, statistically, can be codified as Bayesian updating of the posterior probability of the trait. The aim of genetic-based predictive models is to augment existing laboratory, imaging, and other clinical data to improve the posterior probabilities (i.e., drive the posterior probabilities toward 0 or 1) of medical traits in a cost-effective manner.

In the context discussed here, predictive models are methods designed to use clinical, analyte, genetic, or other types of data for the purpose of forecasting a medical trait. Predictive models—including those based on genetic markers—are most beneficial when they yield actionable and individualized results. However, they are of reduced value if they only substantially modify medical decisions for an exquisitely small fraction of the patient population. Hence, the ideal genetic-based predictive model for clinical applications (1) substantially modifies the posterior probability of medical traits compared to that obtained from existing clinical assessment and tests—enough to enable changes in medical decisions and patient management, and (2) impacts a substantial fraction of individuals to whom it is applied and provides improved outcomes. While other considerations are essential, such as more cost-effective care and the ease of adoption and implementation of diagnostic tests, it is this concurrent maximization of (1) modifying the posterior probability of the trait in the context of the benefits and risks of the specific medical decisions and (2) broad applicability that defines an archetypal genetic-based predictive model. For example, genetic testing of *CFTR* mutations for cystic fibrosis is successful in that the recessive disease alleles have very high penetrance and the large majority of pathogenic mutations are covered with contemporary panels. Similarly, multi-gene tests for related rare diseases, each with high penetrance, can also serve as useful clinical tests (Rehm, 2013).

In an attempt to develop such predictive models, many have used genome-wide association study (GWAS) results as they are a ubiquitous source of genetic information (Manolio, 2010). Attempts to use genetic information alone have not been as successful as previously hoped, with posterior probabilities that do not approach 1 or 0 and the vast majority of individuals having decidedly intermediate posterior probabilities. A seminal question is the extent to which genetic information can further modify posterior probabilities for those individuals thought to be of intermediate risk using traditional factors. Wray et al. offer an excellent review of the challenges involved in complex trait prediction with GWAS results (Wray et al., 2013).

The discovery of genetic markers for the prediction of medical traits is entirely dependent on the underlying genetic model that gives rise to the trait. That is, the number of loci and the number, frequency and penetrance of predisposing alleles determine both the likelihood of identifying causal markers and the clinical utility of using those markers in a patient population. For example, monogenic disorders such as phenylketonuria, Tay-Sachs, or sickle cell anemia are likely fully penetrant with allele frequencies that are not exceedingly rare; and therefore genetic tests for such diseases have clinical applications, provided that disease avoidance or disease-modifying treatments exist. However, traits like Alzheimer's disease, diabetes, or response to statins have etiologies that remain enigmatic. Whether or not these complex traits follow extremely polygenic modes of inheritance (i.e., weakly penetrant alleles, and several hundreds to thousands of loci), high locus/allelic heterogeneity (having highly penetrant but unique loci and alleles involved across individuals), high levels of epistasis (e.g., genotypic effects that vary based on genetic background or other specific genotypes), ubiquitous epigenetic effects (e.g., methylation patterns, histone acetylation patterns, or transgenerational RNA artifacts affecting the trait), gene-environment interactions, or some combination thereof, directly impacts the identification of predictive markers as well as their utility. GWAS interrogate the common allelic architecture for disease predisposing markers exhibiting low degrees of allelic and locus heterogeneity, whereas sequencing-based studies in families can facilitate the discovery of rare disease-associated variants, but are not optimal for identifying ancestral disease-predisposing alleles. Therefore, it is reasonable to expect that genetic markers from GWAS may modify posterior probabilities across a large segment of the population, but with a muted impact on those probabilities. On the other hand, rare sequence variants, on the other hand, may have substantial impact on the posterior probabilities for specific individuals, but with little widespread effect.

A review of the potential of genetic-based predictive models to change medical practice in the short-term indicates that three areas have shown promise for improving clinical care: cancer genomics, population screening for Mendelian diseases, and pharmacogenetics. These three areas profit from high penetrances of the genetic variants identified to date, though only a fraction of patients benefit from these tests. As these areas emerge from their infancy and additional genetic results accumulate, the proportion of individuals benefiting will likewise increase.

### Prediction using tumor genomics

The advent of genetic testing in tumor cells, through harnessing the throughput and read depth of next-generation sequencing platforms, has enabled detailed and clinically actionable molecular pathology genetic tests for numerous cancers. Multiplex sequencing-based assays for biopsies compared to normal tissue are now available and have demonstrated usefulness in augmenting many clinical decisions. The utility of these tests relies on the clear relationship that has been delineated over the past two decades between specific driver mutations, treatment variants and cancer progression, and drug selection (Liaw et al., 1997; Paez et al., 2004; Agrawal et al., 2011; Walter et al., 2012; Kandoth et al., 2013; Vogelstein et al., 2013). Intratumor (Gerlinger et al., 2012) and single-cell sequencing methods (Navin et al., 2011) offer the possibility of inferring the evolutionary history and driver mutations in clonal expansions of cancer cells. These techniques have been successfully applied to several cancers with excellent prognostic utility, for example, kidney cancer (Xu et al., 2012). For well-defined activating mutations such as those within *BRAF* (Loupakis et al., 2009; Borras et al., 2011), *KRAS* (Linardou et al., 2008) and *EGFR* (Lynch et al., 2004), the posterior probability of efficacious treatment selection is also high. Indeed, there seems to be a clear path to incorporating panels of well-defined oncogenesis, metastasis, and drug response variants through next-generation sequencing of tumors. Baylor College of Medicine, one institution among several offering a number of clinical genetics tests, has developed a Cancer Gene Mutation Panel through next-generation sequencing that investigates 2855 known mutations within 50 cancer-associated genes for clinical testing (http://www.bcm.edu/cancergeneticslab/test_detail.cfm?testcode=9705). Other efforts include the UCLA Clinical Genomics Center (http://pathology.ucla.edu/body.cfm?id=105), the Emory Genetics Laboratory (http://genetics.emory.edu/egl/), and the Washington University School of Medicine (http://gps.wustl.edu/). Identification of a small number of specific mutations enables selective treatment courses to be taken with higher expected efficacy, albeit often with limited duration of effect due to the development of drug resistance, an expected consequence of monotherapy. For example, in this Baylor panel *BRAF* mutations are targeted, where treatment with vemurafenib and dabrafenib has demonstrated *BRAF* Val600-specific metastatic melanoma antitumor activity (Jang and Atkins, 2013). Over the past five years, somatic cell and tumor genomics has provided remarkable insights into the molecular pathobiology of cancers. This rapidly progressing field continues to accumulate examples of improved treatment resulting from these genetic discoveries.

### Prediction in screening for mendelian disorders

Equally impressive has been the progress in interrogating very highly penetrant alleles in population-based screens, particularly in newborns. Next-generation sequencing has enabled rapid, cost-effective multiplex assays that require little DNA. Given the high positive predictive value of these variants and the ability to modify clinical treatment in many of these Mendelian disorders, genetic-based prediction in this area is an efficacious addition to medical practice. For example, Saunders et al. recently showed the feasibility of screening for monogenic diseases across the genome within 50 h in a neonatal clinical setting (Saunders et al., 2012). Importantly, infants identified as having pathogenic genotypes (e.g., Kwan et al., 2013; Stefanutti et al., 2013) can receive appropriate treatment while still hospitalized, often avoiding life-threatening complications. Comprehensive genetic testing may preclude emotionally and financially costly pediatric odysseys (Kingsmore et al., 2011). In addition, the application of high-throughput sequencing approaches to clinically important, expansive gene panels can reliably identify known inherited pathogenic variants and new germline mutations that are potentially pathogenic, thereby driving effective early screening (Kurian et al., 2014).

### Prediction in pharmacogenetics

Pharmacogenetics is the third area in which genetic variants can enable physicians to differentially prescribe certain medications to individuals to avoid adverse events or to modify dosing. The importance of these genetic variants in avoiding adverse drug reactions is underscored by FDA black-box warnings (http://www.fda.gov/Drugs/ScienceResearch/ResearchAreas/Pharmacogenetics/ucm083378.htm), as well as by recommendations of other groups (https://www.pharmgkb.org/). For example, individuals carrying the HLA-B^*^5701 allele are warned against taking abacavir (Mallal et al., 2008), dapsone-treated patients with certain *G6PD* variants are at higher risk for hemolysis as are patients receiving primaquine, many sulfonamides, nitrofurantoin, acetanilide, niridazole, and naphthalene (Cappellini and Fiorelli, 2008), and the beta blocker propranolol can cause adverse reactions in those with variants conferring compromised CYP2D6 function (Cascorbi, 2003; Samer et al., 2013). In all, the FDA currently lists 155 pharmacogenetic warnings across numerous therapeutic areas. Again, the validated, high positive predictive value of these pharmacogenetic variants makes immediate clinical utility possible, if not immediately actionable. Clinically useful genetic variants underlying other pharmacogenetic traits, such as differential response to many lipid-modifying medications, metformin, and anti-TNF therapies, still remain largely abstruse.

Importantly, the setting of clinical application of genetic tests is critical to the usefulness of genetic-based predictive models. Traits, including drug response and adverse reactions, that are (1) otherwise easily diagnosed, or (2) for which disease management would not change with the results of the predictive model, are poor candidates for these predictive models. So, results from the use of genetic-based predictive models must serve as a cog in the health management machinery and clearly satisfy an unmet medical need. For example, genetic-based predictive models are unlikely to play a useful role in diagnosing a bone fracture. Similarly, Kimmel et al. recently showed that even though genetics can fairly accurately predict warfarin sensitivity, this information offers no benefit over clinical management of warfarin dosing to achieve therapeutic range (Kimmel et al., 2013). The setting of medical care also plays an important role: nearly half of all patients are not treated in coagulation centers, leaving the question of how diagnostic genetic testing would fare in those environments.

Why is it that these three above areas have enjoyed more success in applying genetic information to clinical practice than other applications, such as prognosis of complex diseases? In large part, the answer lies in the relatively low complexity of the genetic architecture behind these medical traits. The propagation of cancer cells, tumor survival and metastasis are promoted by specific mutations that wield strong effects on promoting clonal expansions: driver mutations. Different driver mutations accomplish this task in different ways, but each driver mutation has profound effects on cellular metabolism, mitosis, and proliferation. Because the effects of these driver mutations are profound and characteristic of specific molecular pathophysiologies, it is not surprising that they are reasonably predictive of disease trajectory and chemotherapy response. Similarly, provided that the false positive rate of prognostic tests is low, population-based screens for Mendelian disorders have been a useful addition to modern medical practice because the penetrance of such traits is typically complete or nearly complete. That is, aside from the measurement error rates, the prediction of disease given a positive genetic test is accurate and reliable. Finally, although not as definitive as Mendelian disorders, pharmacogenetic effects identified to date testify to reduced complexity of these traits. This is particularly true of extreme adverse events (e.g., FDA black box warnings) and response with those drugs having highly targeted substrates. In contrast, therapeutics with multifold actions, such as statins or metformin, have exhibited much more recalcitrance to genetic dissection.

In contrast to the above-mentioned areas, currently the prediction of common diseases presents a considerable challenge. Most common diseases have been relatively reluctant to reveal a large fraction of the genetic component of their etiologies (Manolio et al., 2009). Several studies of complex diseases have shown little improvement to disease prediction when adding genetic data to already established disease risk factors (e.g., Thanassoulis and Vasan, 2010; Bao et al., 2013; Muhlenbruch et al., 2013); and, even if statistically significant, the models incorporating genetic information may not be clinically useful (Husing et al., 2012). While there are several instances of important, influential markers that have been discovered in some common diseases, such as *APOE* in Alzheimer's, *ARMS2* and *CFH* in age-related macular degeneration (AMD), and numerous alleles in the MHC region for autoimmune and inflammatory diseases, many genetic linkage results are the result of multiple infrequent alleles and most replicated markers from GWAS have modest effect sizes. In combination, the replicated disease susceptibility alleles discovered thus far have yet to demonstrate substantial prognostic utility. That said, there are encouraging exceptions: the combined effect of the multiple identified loci for AMD or Crohn's disease may offer some clinical utility in selected circumstances. AMD is a leading cause of compromised vision and blindness, and individuals at heightened risk for AMD can benefit from more frequent eye exams and early treatment to curb the likelihood of permanent ophthalmic damage. Administration of anti-VEGF monoclonal antibodies have shown efficacy in exudative AMD treatment (Fung et al., 2007; Heier et al., 2012). Recent GWAS studies in AMD have demonstrated that the 19 top AMD risk loci are estimated to explain between 15 and 65% of the genetic portion of the variance in the phenotype (the proportion depends on the assumption of AMD prevalence being between 0.01 and 0.10). This set of SNPs also generates an area under the ROC curve (AUC) of 0.74 (Fritsche et al., 2013), meaning that if you choose pairs of people at random, one with and one without AMD, and used their SNP data, the one with a higher probability of AMD would in fact be the one with AMD 74% of the time (Berrar and Flach, 2012). Incorporation of other known risk factors, such as age and smoking, further improves this prediction. It is possible that other measures, including positive and negative predictive values or those based on posterior probability distributions, could provide better insight into clinical utility. Another promising area is the use of all genetic variants genotyped in a genome-wide array to construct predictive models, rather than restricting the markers to those that are strongly associated with the trait. Purcell et al. investigated the use of thousands of common alleles in predictive models for schizophrenia and bipolar disorder, demonstrating an increase in the proportion of the maximum variance in these traits explained as the trait-association significance level was relaxed (Purcell et al., 2009). In addition, analysis of the Wellcome Trust Case Control Consortium data for Crohn's disease appeared to indicate that expansion of the number of SNPs in a predictive model over just those reaching genome-wide significance improves the model performance (Kooperberg et al., 2010). These are interesting observations and consistent with results from Yang et al. (2010) and Lee et al. (2011) describing the rather dramatic increase in the proportion of heritability explained with all GWAS SNPs compared to top SNP findings. Wei et al. offer a recent example of harnessing this effect for Crohn's disease where expanding the number of variants and using advanced machine learning techniques increased predictive accuracy (Wei et al., 2013). With greater resolution, Yang et al. (2011) showed that the length of chromosomes is linearly correlated with the percentage of the variance attributable to a variety of phenotypes, including von Willebrand factor, height, BMI and QT interval. However, both theoretical and applied work appears to show limited utility of including more than a few hundred SNPs in commonly-used predictive models (Wu et al., 2013; Warren et al., 2014). Nonetheless, methods that exploit the whole genome for disease prediction, such as extensions to Best Linear Unbiased Prediction (BLUP), continue to develop and may improve accuracy metrics for both binary disease and quantitative traits (Zhang et al., 2014).

## Utility and methods

Given that for many diseases with effective treatments accurate prediction of potential disease can play a critical role in determining robust clinical care that may avert severe disease or even disease onset, it is essential to characterize the important aspects that produce useful predictive models. For traits with polygenic etiologies, methods must be used to combine signals from multiple genetic markers together into a cohesive metric for prediction (Wimmer et al., 2013). Seven main considerations when doing so are: (1) which genetic markers are to be included in the predictive model—i.e., feature selection, (2) the frequencies of the susceptible/protective genotypes at each selected marker, (3) the strength of the correlation between the genotypes at each marker and the predicted trait, (4) the interactions between the effects sizes of different genetic markers, (5) the prevalence of the trait being predicted, (6) how the genetic data are envisioned to integrate into clinical practice in combination with non-genetic tests, and (7) a determination of the robustness of the prognostic signal across multiple populations, including those with varied ancestries. Over the past decade, several methods have been proposed to accomplish these tasks, including genetic risk scores, various types of regression-based approaches, Bayesian networks, and other machine learning methods. Importantly, polygenic disease-prediction models may serve as instrumental variables for Mendelian randomization analyses in the investigation of the causal role of genetic-based predictors in disease (Burgess and Thompson, 2013).

### Feature selection

Feature selection refers to the decision about which genetic variants are most effective in determining the medical trait and should therefore be included in a predictive model. For example, it would seem reasonable to include SNPs in the *CFH, ARMS2, C3*, and *C2/CFB/SKIV2L* regions in a model predicting AMD because the evidence for correlation between AMD and these variants is both substantial and well-established. Further, selection of these variants for inclusion in a predictive model would be prioritized over other variants with little or no evidence of utility in AMD prediction. Jakobsdottir et al. have investigated the properties of individual disease-susceptibility SNPs, showing that SNPs with highly significant odds ratios may be insufficient to classify individuals (Jakobsdottir et al., 2009). There are several different methods that can be employed. For a general review see Guyon and Elisseff (2003). Care must be taken when internal validation techniques are applied to datasets, as the feature selection must be incorporated in the internal validation routine. Ideally, feature selection should be replicated in an independent sample set. Approaches based on stepwise selection of features are popular. The performance of models constructed based on a stepwise selection can be evaluated based on model fit, accounting for the complexity of the model—the Akaike and Bayesian information criteria are examples of measures to do this (Akaike, 1974; Schwartz, 1978). Aside from purely statistical and computational approaches, use of biological information can improve the selection of genetic markers. By integrating information from numerous decades of biochemistry, molecular biology and cellular physiology—the direct phenotypes of genetic variants—one can construct predictive models weighted toward those variants segregating in functionally relevant regions in an effort to improve the robustness of the model and ease of application to related phenotypes. For example, if one is generating a genetic-based predictive model for Crohn's disease response to IL-17 monoclonal antibody therapy, higher prioritization of variants within IL-17-related genes or those polymorphisms that are known to modify T-helper cells expressing IL-17 (Th17) activity may provide complementary information and yield a higher likelihood of the test having utility when applied to other populations or related phenotypes.

### Genetic risk scores

Genetic Risk Scores (GRS), determined simply on the basis of published GWAS results, are among the simplest methods employed for genetic prediction. The majority of these approaches construct the predictive model based on the sum of predisposing genotypes that each individual carries, either unweighted or weighted by the effect size of the specific predisposing genotypes. The essential approach is to take a weighted sum of risk alleles, choosing the risk alleles based on those found to be genome-wide significant in a recent meta-analysis (e.g., for BMI, see Speliotes et al., 2010). Weights are determined for each risk allele by the β estimates from the meta-analyzed GWAS. Unweighted GRS treat each risk locus equally. To illustrate the weighted GRS approach, assume that k SNPs are known to be genome-wide significant and further assume that the corresponding β weights from the GWAS are denoted as *w_i_* for the *i*th SNP. Then the be calculated as: $GRS=\sum_{i}^{k}w_{i}R_{i};$ where *R*_i_ is the number of risk alleles at the *i*th SNP. Speliotes et al., using 32 confirmed obesity-associated loci, showed the distribution of the weighted number of risk alleles across the population used in the Atherosclerosis Risk in Communities (ARIC) study, and presented a corresponding AUC for the GRS (Speliotes et al., 2010). Although significantly different from that expected under the null, the AUC for this example was exceedingly modest (0.515), where flipping an unbiased coin would be expected to have an AUC of 0.500. In another example, Ripatti et al. developed a genetic risk score based on 13 SNPs discovered to be associated with coronary heart disease, myocardial infarction or both, in seven reports (Ripatti et al., 2010). For each individual, the effects of these SNPs were combined by summing the number of risk alleles and the resulting risk score was partitioned into quintiles for the purpose of creating a categorical variable. Comparing extreme quintiles, the authors found roughly a 1.7-fold increased risk for coronary heart disease in the top risk quintile compared to the lowest risk. The genetic risk score did not show a significant effect of the net reclassification of individuals over traditional risk factors and family history. The combined genetic effect was able to slightly improve the risk classification of those individuals who were previously thought to have intermediate risk as determined by traditional risk factors, but may not have strong clinical utility. Increasing the number of informative genotypes and/or the traditional risk factors may improve the prognostic performance of GRS. Other applications, including age-related macular degeneration, exhibit more promising performance (Grassmann et al., 2012; Seddon et al., 2014).

### Regression methods

Regression methods, familiar tools for constructing prediction models for both dichotomous and quantitative traits, can lead to more general predictive models than simple GRSs. One of the first reports of a cohesive method using multiple replicated markers under a general logistic regression model was developed by Yang et al. (2003). Yang and coworkers proposed using a general logistic regression model to estimate the ratio of the probability of the genotype information given disease to the probability of the genotype information within the non-diseased population. Incorporation of covariates and interaction effects are possible with this generalized form. Currently, regression is still commonly used for disease prediction. For example, a search of PubMed revealed 10 articles published in 2013 which applied regression methods for the prediction of a variety of diseases, including cerebrovascular disease, age-related macular degeneration, and hypertrophic cardiomyopathy (Abraham et al., 2013; Borque et al., 2013; Gruner et al., 2013; Harada et al., 2013; Mondul et al., 2013; Romano et al., 2013; Schellekens et al., 2013; Sharma et al., 2013; Tsai et al., 2013; Uddin et al., 2013). In addition, extensions including regression of the whole genome using a Best Linear Unbiased Prediction method (G-BLUP) can produce more highly predictive models (de Los Campos et al., 2013). Importantly, Yang et al. (2009) pointed out that one should not rely on point estimates alone, but also provide a measure of the uncertainty of the risk estimates. Risk estimates depend on a variety of parameters, each of which may be estimated with some uncertainty. Cumulative uncertainty across all estimated parameters leads to uncertainty of the risk estimates.

There are several modeling assumptions made when applying either linear or logistic regression but, in the specific application area of genetics, the following concerns should be emphasized. First, multicollinearity between nearby markers is usually a serious concern. For markers in high linkage disequilibrium with each other, it is common to select the variant with the lowest *p*-value for inclusion in the model. Principal component regression is another useful way to address concerns arising from multicollinearity. For example, Gauderman et al. found that this approach performs well when applied to a single candidate gene (Gauderman et al., 2007). Another concern is marker-marker interactions. For parsimony, it is common practice to ignore interactions. Interaction analysis is not easy to conduct and can be computationally intensive. Furthermore, substantially larger sample sizes are typically needed to detect interaction effects than are needed to detect main effects. However, ignoring interactions may underestimate genetic effects, and improvements in the understanding of interactions would be expected to improve genetic risk prediction models (Thanassoulis and Vasan, 2010). Missing data are commonly problematic since genotype success rates are never perfect (Kim and Misra, 2007). One strategy is to drop samples with missing data (Schwender and Ickstadt, 2008). Otherwise, when possible, imputation can be a useful solution for “filling in” missing data (Yuan, 2000).

Usually, for presence vs. absence of disease phenotypes, a predictive model is first developed by analyzing a case-control dataset, and then applied to a particular population. To provide risk estimates that are calibrated to that particular population, an adjustment which depends on the case to control ratio must be made to the intercept term of the case-control regression model (Yang et al., 2003).

Many studies, but not all (Warren et al., 2014), indicate that risk prediction would be more accurate if more predictors could be added in the risk model (De Jager et al., 2009; van Dieren et al., 2012). But the confidence interval (CI) of the risk estimate is often not considered in the evaluation of the risk model. When the model is built using regression in a meta-analysis of many case-control datasets, confidence intervals are often not even estimated.

Provided it is unbiased, a more precise risk estimate with a smaller CI from a model with fewer predictors is better than a less precise risk estimate with a larger CI from a model with more predictors (Shan et al., 2013). To compute the CI for the risk estimates from a meta-analysis, each individual study in the meta-analysis should do a joint analysis and return coefficient estimates and the variance-covariance matrix for the coefficients. Then, these can be combined to estimate the overall variance-covariance matrix and a precise CI for the risk estimates. Goddard et al. developed a method that derives an empirical CI combining all relevant sources of variation in disease risk (Goddard and Lewis, 2010; Crouch et al., 2013).

### Bayesian networks

Bayesian Networks have resulted from the application of advances in graph theory to applied probability and carry a high degree of interpretability, along with providing an intuitive framework for obtaining posterior probabilities and the treatment of classification problems (Pearl, 1988; Jordan, 2004). If the features (genetic markers) within the Bayesian Network can be reasonably modeled as being conditionally independent (conditional on the disease trait in our application), then the network is reduced to a highly tractable Naïve Bayes model. Given a set of n genetic markers, using Bayes' rule one can write the posterior probability of the disease trait (PPD), as:

$$PPD_{n}=P\left(D|\cap_{i=1}^{n}G_{i}\right)=\frac{P\left(\cap_{i=1}^{n}G_{i}|D\right)P\text{​}\left(D\right)}{P\left(\cap_{i=1}^{n}G_{i}\right)},$$

where *D* denotes a random variable for the disease trait and n genetic markers are used in the prediction. Under the conditional independence assumption of Naïve Bayes, we can completely factorize the product and, for a binary trait (*D* = 1 to denote disease and *D* = 0 for non-disease), one can re-write the PPD as:

$$\begin{array}{l} PPD_{n}=\\ \;\frac{P\text{​}\left(D=1\right)\prod_{i=1}^{n}P\left(G_{i}|D=1\right)}{P\left(D=1\right)\prod_{i=1}^{n}P\left(G_{i}|D=1\right)\text{ + }P\left(D=0\right)\prod_{i=1}^{n}P\left(G_{i}|D=0\right)}. \end{array}$$

**Figure 1:**
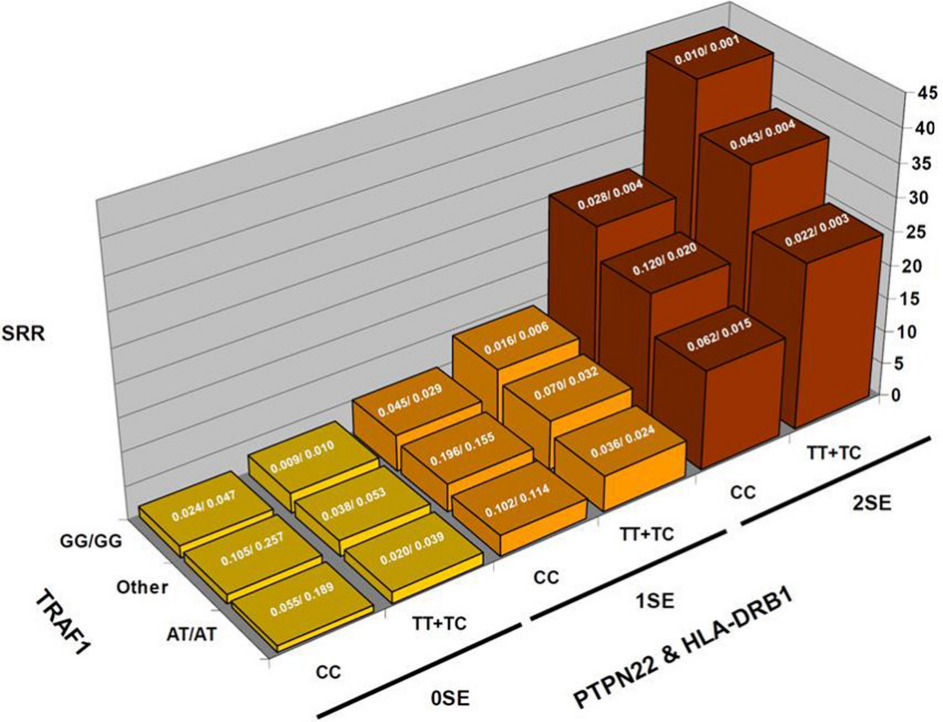
**Rheumatoid arthritis scaled posterior probabilities (SRR)**. Genotype data at three strongly predisposing loci, *HLA-DRB1, TRAF1*, and *PTPN22* are combined and the posterior probabilities calculated for every possible multilocus genotype combination. The prior probability was set to the approximate population prevalence of rheumatoid arthritis, 0.01. The posterior probabilities are scaled such that the lowest RA-risk multilocus genotype was set to a value of 1. The results show a 41-fold variation in posterior probabilities. The expected frequencies of the various multilocus genotype combinations in RA patients/controls are shown at the top of each bar.

To illustrate this type of calculation, Figure 1 shows scaled PPD values for a rheumatoid arthritis study. In this study (Chang et al., 2008), the PPD for every possible three-locus genotype combination at the risk loci (*HLA-DRB1*, the R620W polymorphism at *PTPN22*, and diplotypes at *TRAF1*) was calculated, and scaled such that the smallest value was set to 1; SRR denotes this scaled ratio (Figure 1). While there is substantial variability across the values for different genotype combinations: over a 41-fold difference in predicted rheumatoid arthritis-risk, it is important to keep in mind how these bins are populated with individuals with and without the disease trait (the case-control frequencies given for each combination), for a prognostic loses general utility as intermediate combinations become frequent. In concrete terms, while a 41-fold difference is impressive, only 0.1% of the general population is calculated to carry genotypes producing this level of effect. 3.2% have multi-locus genotypes that generate at least a 21-fold increase in RA risk, and 13.7% carry a multi-locus genotype with >5-fold increase in RA risk (all compared to the lowest category).

**Figure 2:**
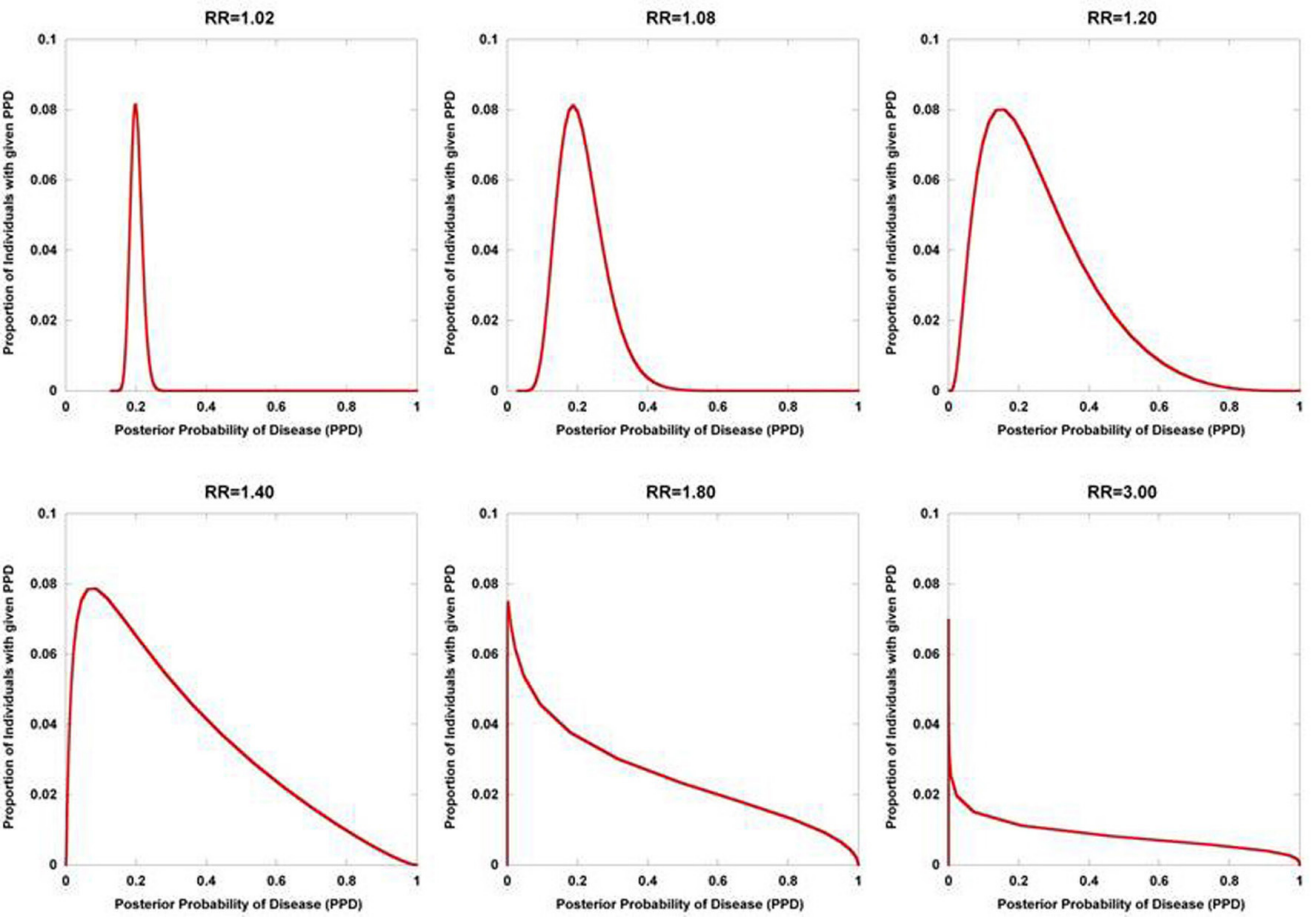
**Posterior probability variation with relative risk**. The density of posterior probabilities of disease (PPD) are shown under a simplified multilocus disease model. The number of independent, disease-predisposing SNPs was set at 500. Relative risk was modeled as being identical for each predisposing SNP. Frequency of the predisposing genotype in controls was set to 0.05 at each SNP. Prior probability of disease was set at 0.20. Naïve Bayes was used to calculate posterior probabilities. The data points only take on discrete values (The densities are composed of discrete values which are connected by lines to produce the curves. While the sum of the discrete values all equal one in each of the curves, the areas under the curves do not), but are presented with interconnecting lines.

**Figure 3:**
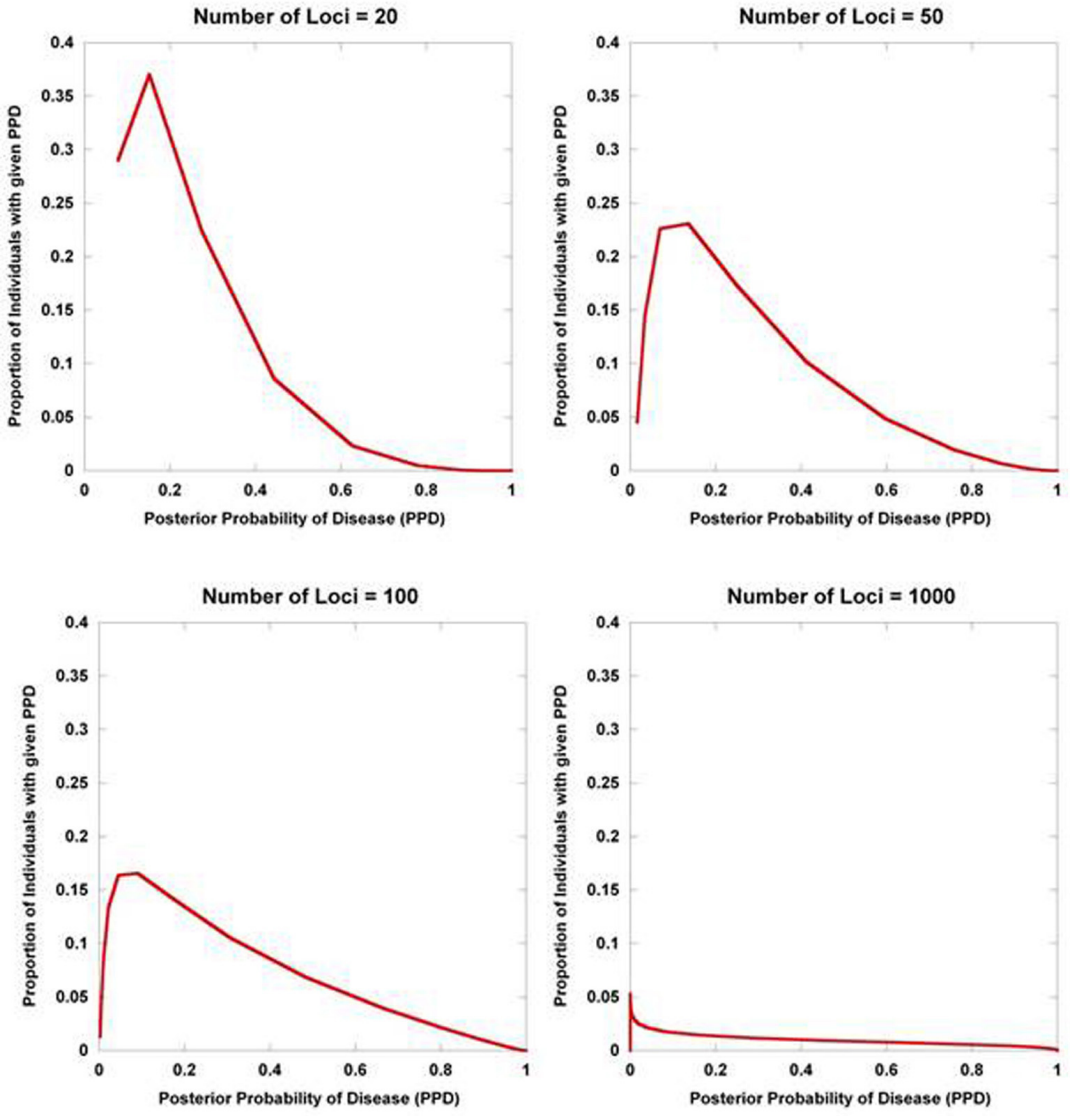
**Posterior probability variation with number of predisposing loci**. The density of posterior probabilities of disease (PPD) is shown under a simplified multilocus disease model. The relative risk of each independent, disease-predisposing SNP was set to 2.0. Prior probability of disease was set at 0.20. Frequency of the predisposing genotype in controls was set to 0.05 at each SNP. The number of predisposing loci was increased from 20 to 1000. Naïve Bayes was used to calculate posterior probabilities. The data points only take on discrete values (the larger number of loci have many more data points reflecting the larger number of possible multilocus genotype combinations), but are presented with interconnecting lines.

Figure 2 displays the results from a simplified model. Five hundred disease susceptibility SNPs, all having equal effect sizes and genotype frequencies, were modeled. A prior probability of disease was set to 0.20 and the predisposing genotype frequency in the general population was set to 0.05 for all 100 SNPs. As expected, for very small effect sizes the number of individuals calculated to have posterior probabilities close to 0.20 is high and rapidly tails off. However, for larger effect sizes, there is an accumulation of individuals with posterior probabilities closer to 1 and 0. Interestingly, even with quite considerable effect sizes (for high frequency alleles) much of the density still resides in the intermediate region between 0.10 and 0.90. If we explore the dynamics as the number of loci is increased, so does the variance in the posterior probability of the disease trait (Figure 3).

Diagnosis or prognosis of disease traits with genetic information are classical problems of classification and clustering within machine learning. Hence, numerous machine learning methods, such as neural networks, support vector machines, and random forests can be applied to these types of data sets. Currently, the use of these methods to address problems using gene expression is arguably more advanced than the analogous methods applied to DNA variation data.

### Quantifying prognostic utility

**Figure 4:**
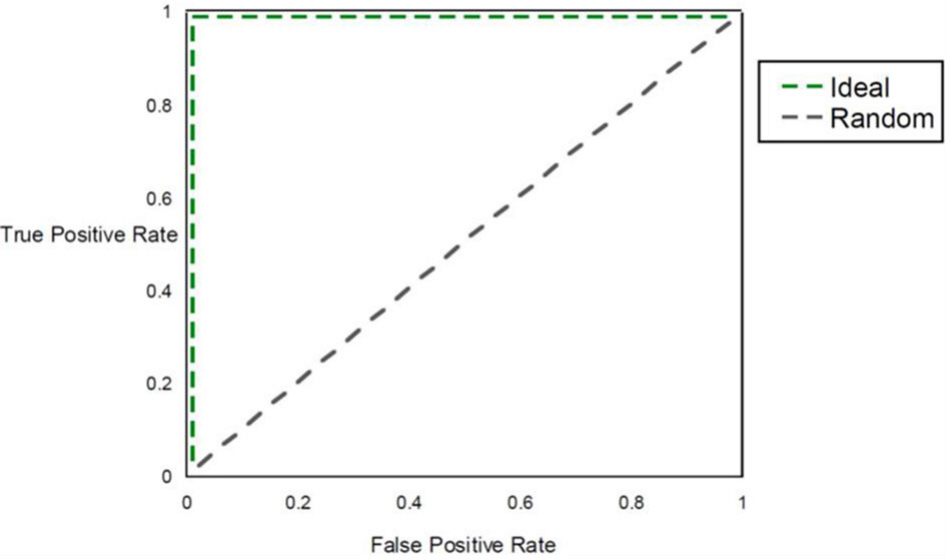
**AUC**. The figure shows the ROC curve and corresponding area under the ROC curve (AUC). The expected patterns under two extreme scenarios are shown: an ideal diagnostic scenario and the pattern expected using random predictions.

Within a population studied, once each individual is (1) assigned a score for a risk metric, (2) assigned a posterior probability, (3) clustered or (4) classified, a method for assessing prognostic utility is required to quantify the usefulness in clinical practice. The most common method used is the area under the ROC curve, or AUC. However, although this metric is useful to assess discrimination, it is not the appropriate measure to assess a predicted probability (Cook, 2007). Graphically, the ROC curve is a plot of the performance of the predictor in a space defined by the sensitivity (true positive rate) and 1—specificity (false positive rate). Varying the threshold of calling a result positive or negative, a curve can be produced for the predictive model. The AUC is the integral of the curve. For a completely non-informative predictor, the AUC is 0.50, with larger values (up to unity) indicating improved prognostic utility (Figure 4). While useful, sensitivity and specificity are probabilities conditional on the state of the phenotype trait. One may want to consider metrics that have differential performance with the prevalence of the disease trait. Indeed, all other diagnostic factors being equal, a physician should be more prone to diagnose an individual with a more common phenotype than an exceedingly rare one, because the *a priori* likelihood of the disease being the common phenotype is higher than the likelihood for the rare phenotype. Therefore, use of positive and negative predictive values (PPV and NPV) may be more useful in the clinical setting. PPV is defined as the proportion of true positives out of all positive results as determined from applying a diagnostic test. Conversely, NPV is defined as the proportion of negative results that are indeed truly negative. However, a direct ROC analog of characterizing the tradeoff between PPV and NPV offers challenges. Motivated by this, Pencina et al. suggest that averaging over PPV and NPV may provide an improved metric for characterizing prognostic/diagnostic utility (Pencina et al., 2008). In 2006, a new method for characterizing disease predictions based on proportions of individuals accurately reclassified was presented (Cook et al., 2006). This approach was further developed in subsequent publications, describing the employment of the Hosmer-Lemeshow goodness-of-fit statistic and the net reclassification improvement statistic applied to reclassification categories as predictive measures (Cook, 2007; Cook and Ridker, 2009). The authors applied these approaches to better specify results from cardiovascular risk models.

Another approach would be to characterize the improvement in the distribution of posterior probabilities as compared to the distribution of prior probabilities, where the distribution is taken across all individuals evaluated. The more informative the genetic information becomes, the larger the departure between posterior and prior probability densities. A natural measure for this is the Kullback-Leibler Divergence, which quantifies the departure between two densities (Kullback and Leibler, 1951). Applied to characterizing the improvement in disease prediction following the interrogation of a suite of features such as genetic markers, the Kullback-Leibler Divergence is defined as:

$$D_{KL}=\sum P\text{​}\left(Disease|G_{1},\ldots ,G_{n}\right)log\text{​}\left[\frac{P\text{​}\left(Disease|G_{1},\ldots ,G_{n}\right)}{P\text{​}\left(Disease\right)}\right]$$

where *G_i_* are the random variables describing the states of each genetic marker involved in disease susceptibility, and the sum is over all possible multilocus genotype combinations. *D_KL_* is calculated across the entire population to whom the predictive model is applied. Larger values of *D_KL_* indicate enhanced differences between the posterior and prior probabilities across the population, reflecting the greater utility of the genetic information. Hence, the Kullback-Leibler Divergence concurrently captures both the magnitude of the effect the genetic data have on the posterior probabilities for each individual (compared to the prior) and the proportion of tested individuals exhibiting each magnitude of the effect. Empirical-based calibration of this or other measures of prognostic utility can often be accomplished through using well-studied data sets having standard prognostic tests such as the Framingham population and cardiovascular disease risk score (Wilson et al., 1998; Schrodi et al., 2009).

Another possible method for characterizing would be to define some level of probability that is clinically meaningful for the specific application. That is, define critical levels τ_*pos*_ and τ_*neg*_ such that exceeding these values with the posterior probability of disease provides actionable information for a clinician. Define the conditions:

C_1_: *P(Disease | Genotype Data, other features) > τ_pos_*

C_2_: *P(Disease | Genotype Data, other features) < τ_neg_*.

**Figure 5:**
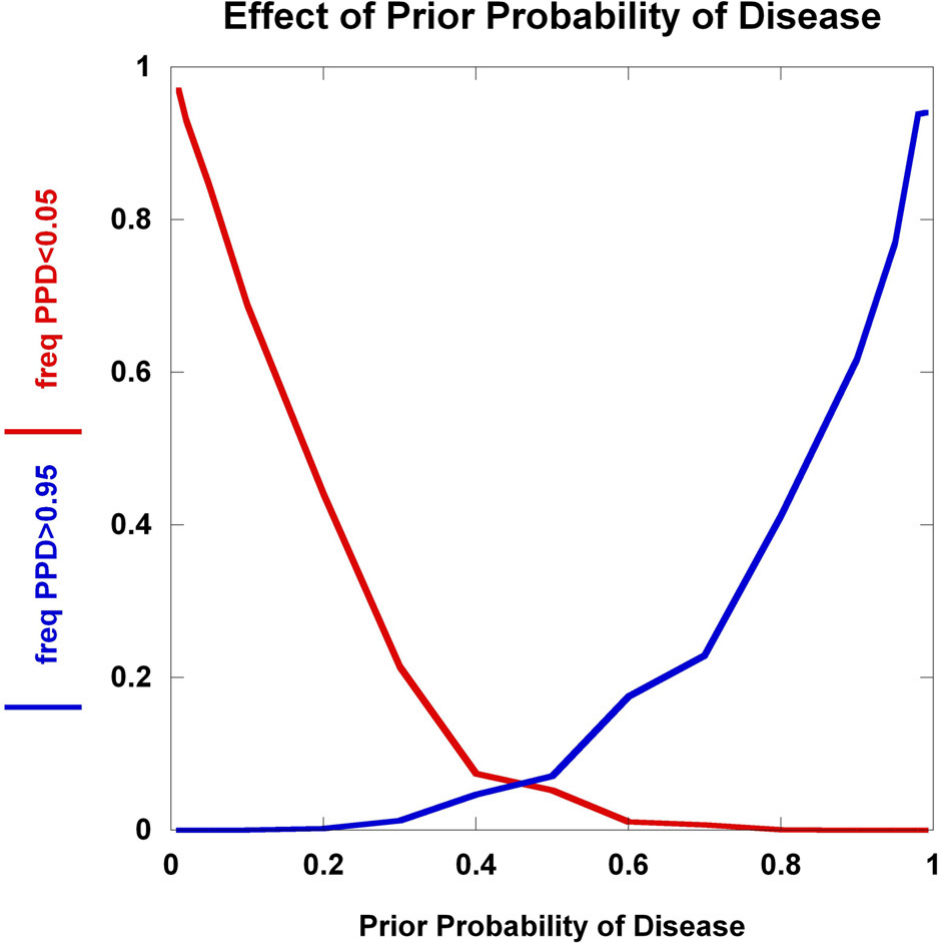
**Effect of prior probability**. The frequency of multilocus genotype combinations exceeding the C_1_ and C_2_ thresholds for posterior probabilities of disease (PPD) (set at 0.05 and 0.95, respectively) are presented as a function of the prior probability of disease. 100 predisposing SNPs were used in the model, each having a predisposing genotype frequency of 5% in controls and relative risk of 2.0.

We explore the dynamics of C_1_ and C_2_ as a function of the prior probability of disease in Figure 5. The collective effect of 100 disease-predisposing SNPs, each with relative risk 2.0 and genotype frequency 5%, is clearly not sufficient to concurrently generate high proportions of individuals who are well-classified as either being likely (C_1_) or unlikely (C_2_) to have disease. At prior probabilities close to 0.50, the majority of individuals do not satisfy either condition C_1_ or C_2_. It is only in the situations where the prior probability is either close to 0 or 1 that large percentages of the population interrogated achieve very high or very low posterior probabilities. Hence, with current results from disease genetics, it seems reasonable to assume that a clinician should already have a strong suspicion either of a disease diagnosis or the exclusion of a disease to warrant the use of SNPs.

**Figure 6:**
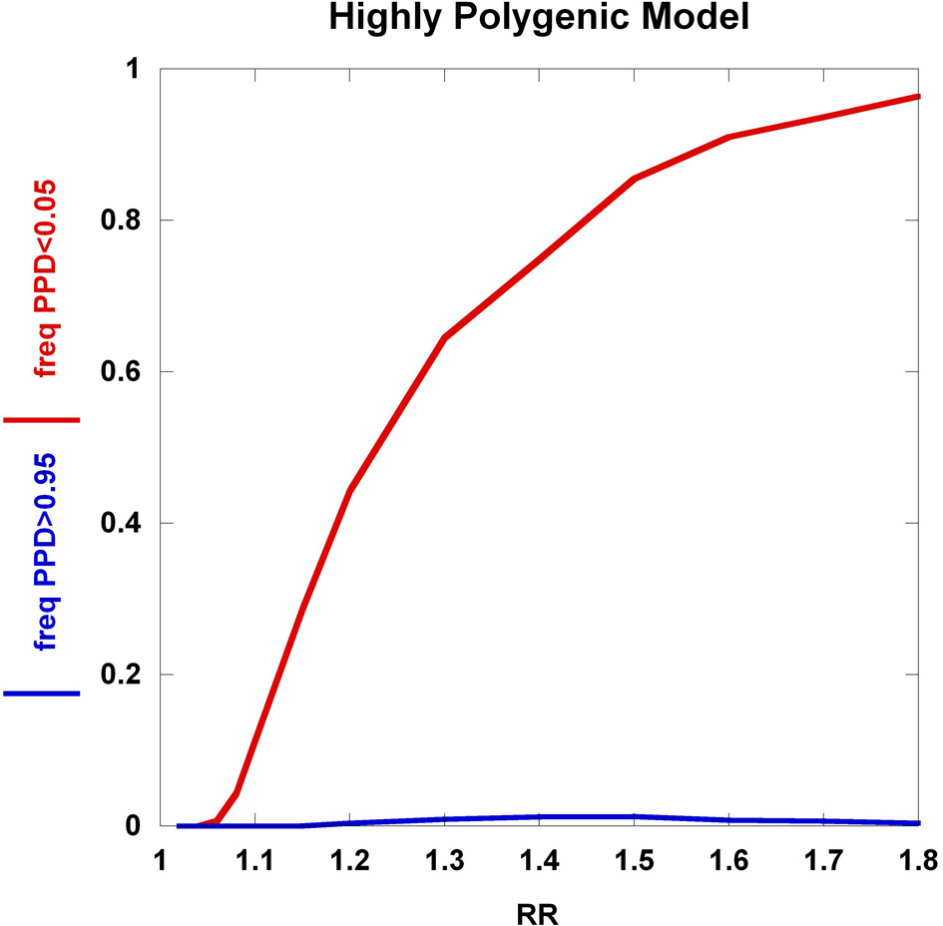
**Highly polygenic model**. The dynamics of the C_1_/C_2_ threshold values under a simplified model is shown as the relative risk of the SNPs varies. The *highly polygenic model* has 1000 predisposing SNPs each having predisposing genotype frequencies in controls equal to 10% and a prior probability equal to 0.20. The relative risk was varied from 1.02 to 1.80.

**Figure 7:**
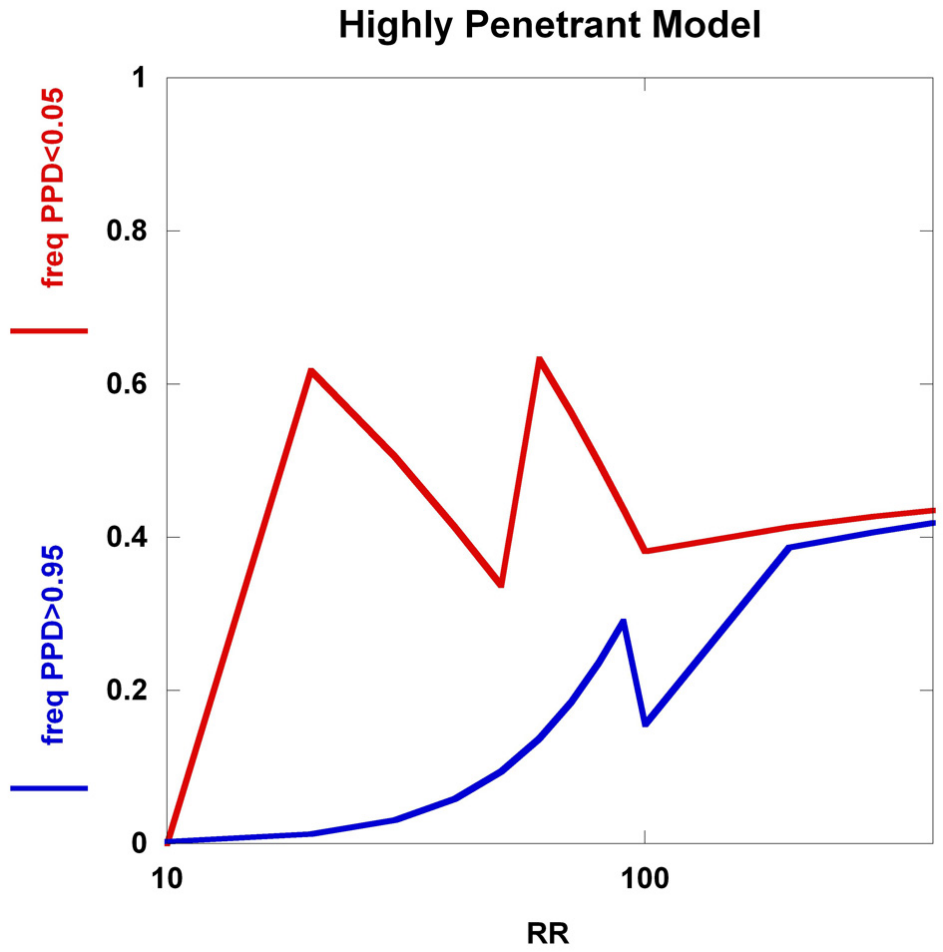
**Highly penetrant model**. The *highly penetrant model* uses 100 SNPs each having a predisposing genotype frequency of 0.1% and also a prior probability of 0.20. The relative risk takes on values from 10 to 400. Although the *highly polygenic model* yields a large proportion of individuals with posterior probabilities below 0.05, the increasing relative risks have little impact on the proportion of individuals with posterior probabilities above 0.95. The *highly penetrant model* shows an overall increase in the proportions of individuals with posterior probabilities below 0.05 and above 0.95, but the patterns are somewhat unexpected (not smooth, nor monotone). These patterns are generated from all predisposing SNVs having identical genotype frequencies and relative risks, coupled with having specific PPD thresholds.

To further explore these prognostic utility patterns, we considered two simplified disease models: a highly polygenic model consisting of 1000 predisposing SNPs, each of appreciable frequency (10% in controls) (Figure 6). We set the prior probability of disease to 0.20. As the relative risk of each SNP is increased from 1.02 to 1.80, the C_2_ condition exhibits a sigmoidal pattern, climbing to over 80% roughly when the relative risk hits 1.45. In contrast, the C_1_ condition peaks at roughly the same relative risk and declines thereafter, but never exceeding 0.02. A typical large GWAS experiment would be well-designed to identify the SNPs with relative risks in excess of roughly 1.1. The collective effect from the 1000 SNPs is not sufficient to overcome the prior probability of 0.20 to promote frequent individual multilocus genotype combinations to exceed the 0.95 threshold of C_1_. That said, the proportion of individuals with posterior probabilities exceeding the C_2_ < 0.05 threshold was much higher. We explored a highly penetrant, rare allele model (Figure 7). We constructed this model with 100 predisposing single nucleotide variants (SNVs) with predisposing genotypes being rare (0.1%), offset by large effect sizes ranging from relative risks of 10 to 400. Sequencing studies generate numerous SNVs. In each graph a single effect size was assumed for all SNVs. Again, the prior probability was set to 0.20. Here, the C_1_/C_2_ dynamics are more complex, with the C_1_ and C_2_ conditions being very sensitive to individual multilocus genotype combinations. Modeling a distribution of SNV frequencies would smooth this type of graph. These are overly simplified cases examined here and the parameter space is vast—additional work in this area would provide useful insights into the properties of prognostics that result from different genetic-based disease models. That said, the proportion of individuals satisfying C_1_ is dramatically higher than under the highly polygenic model. Further, high values of C_1_ and C_2_ occur concurrently. Although much more work is needed to fully explore these dynamics, this observation may give some hope to the usefulness of rare, highly penetrant sequence variants in the context of disease prediction. However, one might have expected that the most common results of GWAS analyses—identification of large numbers of common variants each with small impact on disease risk for common diseases—would be more useful, unless there are other effects operating, such as considerable locus heterogeneity for common diseases.

## Examples

### GWAS data applied to a type 2 diabetes prospective cohort

Type 2 diabetes (T2D) is a common medical condition with rapidly increasing incidence worldwide (Zimmet et al., 2001). This disease is characterized by a multitude of abnormal pathophysiological states involving muted beta cell response, chronic inflammation, and aberrant levels of metabolic markers that ultimately lead to vascular damage, infection, heightened cardiovascular disease risk, and neuropathy (Zimmet et al., 2001). Numerous T2D GWAS have been conducted and have reliably identified new genes and genetic regions involved in T2D susceptibility, albeit with modest effect sizes (McCarthy, 2010). Early prediction of T2D onset and trajectory can be leveraged into improving important medical decisions, including treatment with therapeutics, exercise programs, and diet restriction. It is possible that genetic variants may play a role in improving the prediction of T2D. To test this idea, Shigemizu et al. very recently performed a two-stage study (training and test sets) that resulted in combining nine SNPs with three clinical risk factors (age, gender, and BMI) to develop a predictive model for T2D in a prospective cohort having Japanese ancestry (Shigemizu et al., 2014). The features used in a regression model for T2D-prediction were selected using a Bayes Factor and lasso method. From both genetic and clinical risk factors, the resulting predictive model showed reasonable AUC values in the independent test set (AUC = 0.808). Further, where the sensitivity and sensitivity were concurrently maximized, the model yielded a PPV and NPV of 77.8 and 73.8%, respectively. Although the selected SNPs did add to the diagnostic performance of the prediction model, they only did so in an incremental fashion. The model using SNPs, interactions, and clinical risk factors exhibited a 1.5% increase in the AUC over the clinical risk factors alone. Perhaps the discovery of additional T2D risk variants from sequencing efforts, rarer exome variants, extensive epistatic models, and/or undiscovered epigenetic factors will drive further work in this area to markedly improve the performance of T2D predictive models using heritable information. Until then, there may be greater gains through the use of dynamic markers like metabolite profiling and proteomics. Alternatively, exploration of prediction within T2D subgroups may offer a more fruitful avenue of inquiry.

### Stroke prediction using genetic risk scores

Stroke events are major contributors to mortality and morbidity, constituting the fourth leading cause of death in the United States. Accurate prediction of ischemic stroke risk would enable medical interventions to at least partially remediate stroke occurrence and the resulting brain damage. Very recently, two large studies (Ibrahim-Verbaas et al., 2014; Malik et al., 2014) have been published evaluating risk models constructed from a number of stroke and related phenotype-associated GWAS SNPs. The results were consistent with GRSs achieving statistical significance, but adding little in diagnostic utility to clinical features, as measured by AUC. Ibrahim-Verbaas et al. evaluated the performance of a 324-SNP GRS in four population-based cohorts totaling over 22,000 individuals in an effort to improve the discrimination of ischemic stroke over that generated from the Framingham Stroke Risk Score Model, age and sex. The SNPs were selected based on association with stroke-related phenotypes and a GRS constructed using weights from the regression model used to test the disease association. ROC curves from the results for the study show that the weighted GRSs do not substantially add to the AUC over that achieved by the Framingham Stroke Risk Score and sex—the improvement in AUC from the GRS was approximately 0.02 for all stroke as well as for ischemic stroke alone—although the AUC improvement was statistically significant. The authors also examined the impact of the GRS on the net reclassification index, showing statistically significant, but incremental improvement. Concurrently published in the same issue of *Stroke*, Malik et al. presented similar results for their stroke GRS performance in comparison to clinical features using overlapping samples (Malik et al., 2014). The study showed increased stroke risk across quintiles of their GRS, obtained from an analysis of independent samples from the Wellcome Trust Case Control Consortium 2 and the METASTROKE consortium. Slightly under a 1.5-fold increase in risk was found comparing the top quintile to the middle quintile, and a >2-fold increase comparing the lowest quintile to the top quintile. No significant improvement in the net reclassification was observed and the ROC curves with and without the GRS are virtually superimposable for a sample set composed of a clinical trial-based derivation sample set and the replication sample set.

### Prediction using biobank data

Current efforts to discover and employ genetic risk predictors across multiple health care systems include those of the Electronic Medical Records and Genomics (eMERGE) Network (Gottesman et al., 2013). The eMERGE Network has supported large-scale genotyping efforts in biobanked DNA samples linked to electronic medical records. As such, a repository of genome-wide genetic data can be interrogated with respect to a vast amount of clinical information. One use of these data is to investigate how sets of genetic markers can stratify sample sets for the purpose of performing historical prospective studies. By analyzing longitudinal data, one can specify the sets of individuals to “follow” from a point in time to test for association with various medical traits. In doing so, one can perform a prospective study relating genotypes to the accumulation of various medical outcomes and laboratory values. This is an excellent venue for evaluating genetic-based predictive models. For example, suppose one constructed a predictive model for myocardial infarction (MI) with existing literature findings and then assigned a predicted MI risk for each individual. One could then evaluate how the predicted risk was correlated with the actual conversion rate of non-MI individuals to MI disease states. One can also simultaneously perform association testing between any combination of sequence variants and/or GWAS SNPs and prospectively occurring disease, for the purpose of discovering novel genotype-phenotype correlations. Notably, this type of experimental design is less subject to confounding effects when compared to retrospective case-control designs because a cohort-based design is less likely to impart bias from sample selection being correlated with genetic factors. As noted in a 2010 Institute of Medicine “Rapid Learning” document, the hope is that electronic medical records, biobanks and bioregistries will provide evidentiary support for intervention decisions (National Research Council, 2010). Interesting, Lauer and D'Agostino recently suggested that the next disruptive technology in clinical research would be the randomized registry trial (Lauer and D'Agostino, 2013).

Deeply phenotyped biobanked datasets can also be used to redefine disease states. GWAS have highlighted SNPs that are undoubtedly correlated with susceptibility to common diseases but, as we have discussed, the alleles discovered thus far explain only a marginal amount of disease heritability. The reasons for this are the subject of much debate. Resolution of this perplexing problem will likely involve a multitude of discoveries, not the least of which stem from addressing the opaque correspondence between clinical phenotypes and underlying molecular pathologies. Due to reliance on observations of complex, gross physiology in the clinic, it is reasonable to assume that there may be multiple molecular etiologies that map to a single clinical disease state (e.g., estrogen receptor status now meaningfully partitions previously indistinguishable breast cancers and leads to profound changes in the use of Tamoxifen) (Fisher et al., 1988, 1989; Paik et al., 2004). Conversely, single molecular perturbations may have pleiotropic effects (e.g., the rs2476601 SNP in *PTPN22* is strongly associated with several, clinically distinct autoimmune diseases) (Begovich et al., 2004; Bottini et al., 2004; Kyogoku et al., 2004; Velaga et al., 2004; Canton et al., 2005; Criswell et al., 2005). The medical field is accustomed to defining diseases with regard to visual inspection and gross anatomical measurements, and therefore may (1) aggregate disparate molecular pathophysiologies and (2) partition the same molecular processes into different disease classes. Indeed, there is not a one-to-one mapping between clinical assessments of disease and molecular processes. Thus, it seems reasonable to adopt the reductionist stance that redefining disease states and processes in terms of the underlying genetic and molecular variation may significantly aid investigation of disease etiologies. In this way, one can construct phenotype-based predictive models for sets of genetic/molecular information—a reverse genetics approach. Several groups have recently taken this approach to mapping disease genes: Pendergrass et al. used this method to interrogate data from the PAGE network (Pendergrass et al., 2013), Hebbring et al. (2013), have performed similar types of studies in the Marshfield Personalized Medicine Research Project samples, and Denny et al. utilized data from the eMERGE Network (Denny et al., 2013). In these studies, clinical phenotypes are screened in electronic medical record (EMR) systems for association with specific genetic variants with known function (or highly likely to have specific impact on biological pathways)—a method pioneered by Ritchie et al. in a large-scale effort to replicate numerous associations using DNA databanks linked to EMRs (Ritchie et al., 2010). Novel disease associations can be discovered through these “PheWAS” studies. In addition, this “bottom-up” (specific genetic variants-to-phenotype) approach can also be viewed as a starting point for using genetic information to redefine disease states in a classification system that more closely mirrors the underlying molecular pathophysiology. For example, screening diseases within a biobank for association with *IL23R* missense variants uncovers sets of disease phenotypes where aberrant Th17 signaling plays a pathogenic role. Autoinflammatory diseases, including ankylosing spondylitis, psoriasis, and Crohn's disease, would all show a common, core aspect to their molecular pathophysiology. Additionally, partitioning by these same variants allows elucidation of disease subgroups. This reclassification can further enable disease prediction, for the phenotypes predicted would exhibit clearer correspondence with the underlying molecular mechanisms.

### Inflammatory arthritis prediction in the marshfield population

To illustrate how to apply machine learning methods to empirical datasets for the purpose of disease prediction and show some of the difficulties with attaining strong predictive signals from GWAS findings, we present an example of using genetic data and samples from an EMR-linked biorepository for the purpose of distinguishing between inflammatory arthritis conditions.

**Figure 8:**
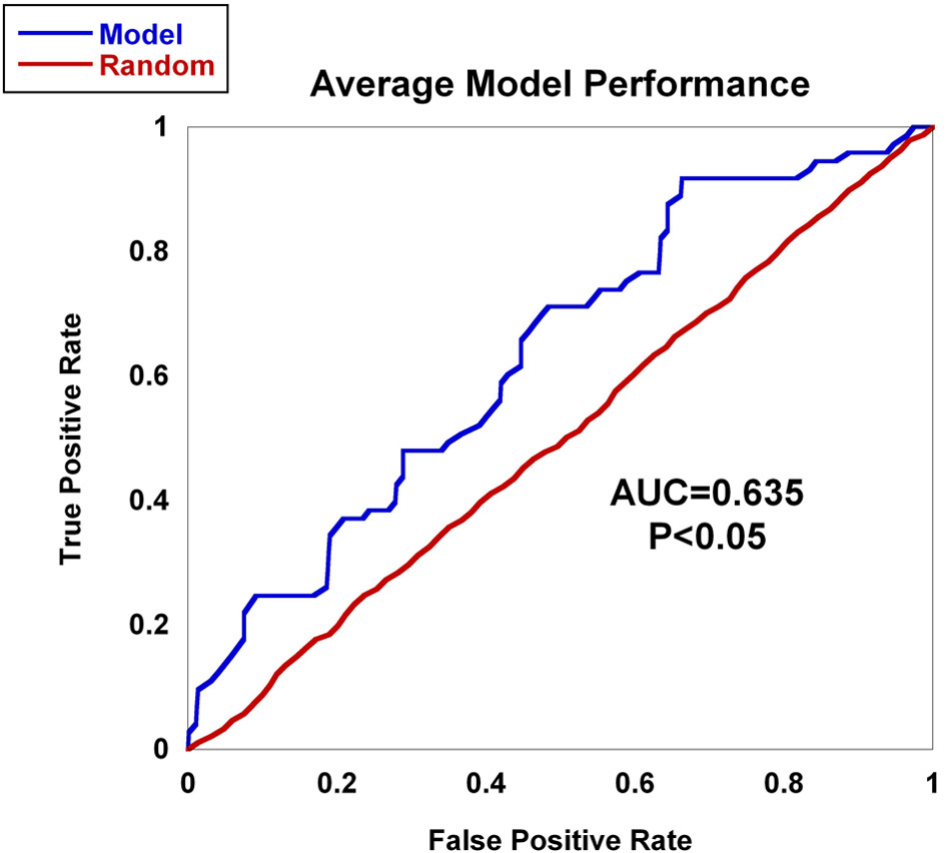
**AUC for inflammatory arthritis prediction study for the Marshfield population**. The Naïve Bayes classifier developed using data from the literature was applied to the Marshfield population of inflammatory arthritis individuals: Rheumatoid arthritis (RA), Psoriatic arthritis (PsA), and Ankylosing spondylitis (AS). The model generated an AUC of 0.635, which was statistically significant via permutation. In addition, performance on randomized sample sets is shown in red, showing an expected null performance.

Worldwide and within the US, inflammatory arthritides are common conditions representing a substantial portion of disabling disease. Early treatment of these conditions can provide substantial benefit in averting disabling articular damage and systemic complications. In general, autoimmune and autoinflammatory diseases such as rheumatoid arthritis and spondyloarthritides have significant heritabilities—a substantial portion of which has been explained by identified polymorphisms, thereby motivating the incorporation of genotype information into prognostic approaches. This study aimed to investigate and characterize the ability of a panel of genetic markers, identified from genome-wide association study results, to classify individuals into the three inflammatory arthritis categories: rheumatoid arthritis, axial spondyloarthritis and psoriatic arthritis (Table 1). Using genotyped samples from an independent sample set from Central Wisconsin (Marshfield Clinic), several machine learning methods were applied to a filtered set of these polymorphisms to classify individuals into the three inflammatory arthritis diseases, blinded to the known disease status. The WEKA software package was used to implement the machine learning algorithms (Holmes et al., 1994; Hall et al., 2009). Accuracy was defined as the proportion of positive and negative classification results that were in fact true. The Naïve Bayes classifier attained the highest average accuracy from 10-fold cross validation on the training set (Table 2). However, when applied to the Marshfield test set, there was a substantial decline in the performance with an average area under the ROC curve of 0.635 (Figure 8). Although the difference between this observed AUC of 0.635 and that expected under the null (AUC = 0.500) is statistically significant, we conclude that additional, orthogonal predictive variables, such as clinical features, circulating cytokine profiles or additional genetic variants, are necessary to build a clinically useful prognostic test for classifying these diseases.

**Table 1:** **Illustration of machine learning methods applied to genetic data: feature selection**.

**Ankylosing spondylitis**	**Psoriatic arthritis**	**Rheumatoid arthritis**
Rs13203464 (HLA-B27)	Rs10484554 (HLA-C)	Rs660895 (HLA-DRB1)
Rs30187 (ERAP1)	Rs20541 (IL13)	Rs2476601 (PTPN22)
Rs11209026 (IL23R)	Rs13017599 (REL)	Rs3761847 (TRAF1/C5)
Rs10865531 (2p15)	Rs2066808 (IL23A)	Rs3890745 (MMEL1)
Rs2310173 (IL1R2)	Rs12924903 (RUNX3)	Rs13031237 (REL)
Rs4333130 (ANTXR2)	Rs4795067 (NOS2)	Rs7574865 (STAT4)
Rs378108 (21q22)	Rs4379175 (IL12B)	Rs548234 (PRDM1)
Rs2297909 (KIF2B)	Rs4982254 (PSMA6)	Rs2327832 (TNFAIP3)
Rs10045431 (IL12B)	Rs13151961 (IL2/21)	Rs1569723 (CD40)
Rs10903118 (RUNX3)	Rs11209026 (IL23R)	Rs11574914 (CCL21)
Rs7720838 (PTGER4)	Rs7720838 (PTGER4)	Rs11172254 (KIF5A)
Rs2058276 (Y-marker)		Rs231804 (CTLA4)
		Rs1160542 (AFF3)
		Rs13151961 (IL2/21)

SNPs were selected for the Naïve Bayes Algorithm to determine inflammatory arthritis categories. Feature selection for the SNPs was performed through a literature search on the three diseases of interest from existing GWAS studies: Ankylosing spondylitis (AS), Psoriatic arthritis (PsA), and Rheumatoid arthritis (RA). The most significant and replicable SNPs were used to construct the entire set. Subsequent evaluation of features used Wrapper Subset Evaluation, ChiSq Attribute Evaluation, Classifier Subset Evaluation, and Information Gain from Attributes methods within the program WEKA. These methods were coupled with a variety of search methods including genetic algorithm-based, Greedy Stepwise Selection, and Linear Forward Search methods within WEKA.

**Table 2:** **Relative performance across machine learning methods**.

**Algorithm**	**Number of SNPs**	**Accuracy (%)**	**10-fold CV accuracy (%)**
Decision tree	33	87	74.1
Neural network	5	76	77.1
Logistic regression	24	78	77.7
Support vector machine	1	73	72.6
K-nearest neighbor	7	77	77.2
Naïve bayes	29	78	77.9

Six Machine Learning algorithms using feature selection and classification were evaluated for accuracy using complied data from the literature in a synthetic training data set. Effect sizes and genotype frequencies were estimated from the literature and incorporated into the synthetic data set to train the algorithms. Accuracy was defined as the proportion of individuals that were correctly predicted (either true positives or true negatives). 10-fold Cross Validation (CV) was performed within WEKA and used as the criterion for selection of an algorithm to use in the test set from Marshfield. Naïve Bayes exhibited slightly higher CV accuracy when compared to other algorithms, with a low amount of overfitting.

## Summary

We have summarized some of the seminal issues in utilizing genetic information in predictive models for disease traits. Currently, the application of genetic-based predictive models to common diseases is, generally speaking, disappointing from both theoretical and empirical lines of evidence. There are some bright spots, including AMD, Crohn's disease, and special applications to selected populations with increased posterior probabilities due to non-genetic factors. Additionally, if the current wave of sequence-based disease gene mapping uncovers sufficient numbers of highly penetrant alleles, then these may provide clinically relevant prognostic utility. Outside of common disease prognostics, tumor genetics, screening for inherited Mendelian disorders, and some pharmacogenetic applications have exhibited the most progress over the past five years. The reasons for this stem from the reduced complexity of the genetic architecture of these traits, yielding extremely high or extremely low posterior probabilities. Certainly, many questions in the field remain. As our understanding of the nature of elements that resolve the missing heritability problem matures, the path to applying predictive modeling methods will become clearer. What needs to fall in place for clinically useful prediction of complex diseases? We speculate that six critical steps will aid this process:

1. Through next-generation sequencing platforms applied to both linkage and association designs, identification of additional susceptibility variants will fully cover the allele frequency spectrum and capture disease-predictive alleles. However, the discovery of rare, highly penetrant risk alleles will be most useful as clinical sequencing becomes widespread and applied earlier in life.
2. As other elements besides DNA sequences are inherited and contribute to phenotypic variance, the interrogation of additional possible contributors to heritability, including DNA methylation patterns, histone modifications, transgenerational effects, and other factors correlated with disease traits, will capture more of the molecularly-defined heritability.
3. Redefining disease phenotypes to more accurately mirror the underlying molecular pathophysiology will be critical in reducing disease complexity and better enable genetic susceptibility mapping. For example, partitioning diseases by molecular subtypes will identify physiological subgroups with clearer correspondence with the underlying genetics. Within the context of research using biobanks linked to medical records, relevant laboratory tests or imaging information, or both, would also be valuable.
4. Considerable progress has been made in the field of machine learning, where robust methods have been developed to select features and use them in predictive models. Applying these approaches to genetic data in combination with existing laboratory tests, imaging data, and other established medical tests will offer the best chance of creating viable prognostics.
5. Metrics that capture prognostic utility in a way that accurately reflects what a clinician requires to inform medical decisions will be developed.
6. The application of disease predictive models to diverse clinical populations will clarify the performance and limitations of proposed predictive models and improve medical practice.

In summary, while prediction will continue to be challenging, future investigations promise to provide a wealth of information, some of which will be clinically useful if considered in the appropriate context.

### Conflict of interest statement

Dr. Steven Schrodi is an inventor on US patents and patent applications, without receiving royalties or any compensation. Dr. John Sninsky an employee of Celera which was recently acquired by Quest Diagnostics. Dr. Sninsky does not receive royalties from patents or separate compensation for patent applications. Daniel E. Weeks holds licensed patents regarding risk prediction for age-related macular degeneration using markers on chromosome 10q26. The authors declare that the research was conducted in the absence of any commercial or financial relationships that could be construed as a potential conflict of interest.
